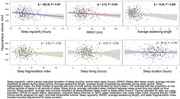# Poor sleep is adversely associated with MRI markers of AD‐specific neurodegeneration

**DOI:** 10.1002/alz.094003

**Published:** 2025-01-09

**Authors:** Hui Shi, Derek B. Archer, Arden Perry, Kimberly R. Pechman, Niranjana Shashikumar, Bennett A. Landman, Timothy J. Hohman, Angela L. Jefferson, Kelsie M Full

**Affiliations:** ^1^ Vanderbilt Memory and Alzheimer's Center, Vanderbilt University Medical Center, Nashville, TN USA; ^2^ Vanderbilt Brain Institute, Vanderbilt University Medical Center, Nashville, TN USA; ^3^ Vanderbilt Memory & Alzheimer's Center, Vanderbilt University Medical Center, Nashville, TN USA; ^4^ Department of Biomedical Engineering, Vanderbilt University, Nashville, TN USA; ^5^ Vanderbilt Genetics Institute, Vanderbilt University Medical Center, Nashville, TN USA

## Abstract

**Background:**

Poor sleep has emerged as a potentially modifiable risk factor for Alzheimer’s disease (AD) and related dementias (ADRD). Few previous studies have incorporated objectively‐measured sleep and structural neuroimaging to understand if poor sleep relates to changes in brain structure. In a cohort of older adults, we investigated actigraphy‐measured sleep health and associations with well‐established magnetic resonance imaging (MRI) markers of AD‐specific neurodegeneration.

**Method:**

Participants from the Vanderbilt Memory and Aging Project (n=390, Mean age: 69.5± 9.3 year; 47.4% women) wore ActiGraph GT9X accelerometers on their wrist daily for 10 days. Data were processed to estimate sleep regularity, wake after sleep onset (WASO), average awakening length, sleep fragmentation index, sleep timing, and sleep duration. 3T brain MRI was used to quantify hippocampal volume and to estimate the McEvoy AD signature (calculated according to published guidelines). We used cross‐sectional multivariate linear regression to assess the associations between individual sleep measures and neuroimaging markers. Models adjusted for age, sex, race, education, APOE‐e4 status, cognitive status, sleep medication use, body mass, index, physical activity, depressive symptoms, and cardiovascular risk. For sleep timing and duration, quadratic terms were added in regression models. Models examining the total hippocampal volume also adjusted for intracranial volume.

**Result:**

In the fully adjusted models, we found abnormal sleep duration (ß = ‐2.35, 95% CI = ‐4.58, ‐0.11) and greater sleep irregularity (ß = ‐0.34, 95% CI = ‐0.62, ‐0.07) were associated with lower AD signature values. Additionally, greater sleep irregularity (ß = ‐262.36; 95% CI = ‐451.71, ‐73.00), greater WASO (ß = ‐2.76; 95% CI = ‐5.30, ‐0.21) and greater average awakening length (ß = ‐74.20; 95% CI = ‐128.47, ‐19.94) were associated with lower hippocampal volumes.

**Conclusion:**

Our results suggest that several measures of poor sleep, including greater sleep irregularity, wake time, and sleep fragmentation, are cross‐sectionally linked to smaller hippocampal volume and smaller brain volumes in regions most affected by AD neuropathology. Longitudinal studies are needed to clarify if poor sleep contributes to brain atrophy over time.